# Differential Association of Visceral and Subcutaneous Adipose Tissue with Treatment Response to Neoadjuvant Chemoradiotherapy in Locally Advanced Rectal Cancer

**DOI:** 10.3390/diagnostics16111624

**Published:** 2026-05-26

**Authors:** Hye Jin Kang, Yong Kyun Won, Eun Seog Kim, Sang Mi Lee, Ik Dong Yoo, Jeong Won Lee, Sun-pyo Hong, Moo-Jun Baek, Dong Hyun Kang, Mee-Hye Oh, Ji-Hye Lee, Si-Hyong Jang, Nam Hun Heo, Ji An Seo, Jae Won Kim, Taesung Ahn, In Young Jo

**Affiliations:** 1 Department of Radiation Oncology, Incheon St. Mary’s Hospital, College of Medicine, The Catholic University of Korea, Seoul 06591, Republic of Korea; 2 Department of Radiation Oncology, College of Medicine, Soonchunhyang University Cheonan Hospital, Cheonan 31151, Republic of Korea; 3 Department of Nuclear Medicine, College of Medicine, Soonchunhyang University Cheonan Hospital, Cheonan 31151, Republic of Korea; 4 Department of Surgery, College of Medicine, Soonchunhyang University Cheonan Hospital, Cheonan 31151, Republic of Korea; 5 Department of Pathology, College of Medicine, Soonchunhyang University Cheonan Hospital, Cheonan 31151, Republic of Korea; 6 Clinical Trial Center, Soonchunhyang University Cheonan Hospital, Cheonan 31151, Republic of Korea; 7 Clinical Research Nurse, Soonchunhyang University Cheonan Hospital, Cheonan 31151, Republic of Korea; 8 College of Medicine, Soonchunhyang University, Asan 31538, Republic of Korea

**Keywords:** radiotherapy, rectal cancer, neoadjuvant concurrent chemoradiotherapy, adipose tissue, treatment response

## Abstract

**Background:** In locally advanced rectal cancer, neoadjuvant concurrent chemoradiotherapy (CCRT) followed by surgery is the standard treatment. Pathologic complete response (pCR) is strongly associated with favorable long-term outcomes; however, reliable pre-treatment biomarkers for predicting treatment response remain limited. This study aimed to investigate the association between CT-derived adipose tissue parameters and pathologic response following neoadjuvant CCRT. **Methods:** A total of 61 patients with locally advanced rectal cancer who underwent neoadjuvant CCRT followed by surgery between 2020 and 2024 were retrospectively analyzed. Visceral adipose tissue (VAT) and subcutaneous adipose tissue (SAT) parameters, including area, index, and mean attenuation (Hounsfield unit, HU), were measured at the L3 level on pre-treatment CT. Patients were classified into favorable (complete or near-complete response) and unfavorable response groups, as well as response and no-response groups. Statistical analyses included independent *t*-tests, chi-square tests, and receiver operating characteristic (ROC) curve analysis. **Results:** In the favorable versus unfavorable response analysis, higher body mass index (BMI), larger VAT area, higher VAT index (VATI), and lower mean VAT attenuation were significantly associated with favorable response (all *p* < 0.05), whereas SAT-related parameters were not. In the response versus no-response analysis, SAT area and SAT index (SATI), but not mean SAT attenuation and VAT-related parameters, were significantly associated with treatment response (all *p* < 0.05). BMI was significantly associated with pathologic response only in the favorable versus unfavorable response group analysis, whereas no significant association was observed in the response versus no-response group analysis. **Conclusions:** CT-derived adipose tissue parameters were differentially associated with pathologic response to neoadjuvant CCRT in locally advanced rectal cancer. VAT parameters, including both quantity and attenuation, were associated with favorable response, whereas SAT parameters were associated with overall treatment response, suggesting compartment-specific roles of adipose tissue in modulating treatment outcomes. While BMI demonstrated a significant association in one subgroup analysis, CT-based body composition analysis may provide more comprehensive and compartment-specific information beyond conventional anthropometric measures, and may serve as a potential imaging biomarker for predicting treatment response.

## 1. Introduction

In locally advanced rectal cancer, neoadjuvant concurrent chemoradiotherapy (CCRT) followed by surgery has been established as a standard treatment strategy, demonstrating improved survival outcomes and increased rates of anal sphincter preservation [1]. Among patients who undergo surgery after neoadjuvant CCRT, pathologic complete response (pCR) is reported in approximately 10–20%, and those achieving pCR demonstrate markedly favorable long-term outcomes, with 5-year disease-free survival rates of 80–90% and overall survival rates of 85–95% [2]. These findings indicate that pCR represents not only a marker of local tumor response but also a key prognostic indicator with implications for long-term survival [3]. Nevertheless, surgical resection inherently carries unavoidable risks, including anastomotic complications, temporary or permanent stoma formation, and long-term functional impairment, which collectively underscore the limitations of surgery-based treatment paradigms [4,5,6]. Furthermore, delayed postoperative recovery and surgery-related morbidity often compromise the delivery of adjuvant chemotherapy (CTx), resulting in completion rates of only 43–73% [7]. Importantly, this limitation persists even among high-risk patients who stand to benefit most from systemic therapy.

To overcome these challenges, total neoadjuvant therapy (TNT)—a strategy in which all systemic CTx is delivered prior to surgery—has been proposed [8]. TNT can be administered as induction CTx followed by CCRT or as neoadjuvant CCRT followed by consolidation CTx, with subsequent treatment decisions tailored according to response, including surgery or non-operative close follow-up [9]. This approach has been shown to significantly improve treatment compliance, facilitate early systemic control of micrometastatic disease, reduce the risk of distant metastasis, and, in selected patients, allow for the deferral or avoidance of surgery [10]. However, with the increasing clinical adoption of TNT, an important limitation has become apparent. Despite the strong association between pathologic complete response (pCR) and long-term survival, pathologic confirmation of complete response is inherently unavailable in TNT-treated patients managed with non-operative strategies. Consequently, post-TNT response assessment relies on clinical complete response (cCR) defined by magnetic resonance imaging and colonoscopic findings, underscoring the growing need for objective biomarkers capable of predicting true pCR prior to treatment.

Meanwhile, previous studies have demonstrated that CT-based body composition parameters, particularly visceral and subcutaneous adipose tissue indices, are associated with survival outcomes and early postoperative prognosis in patients with rectal cancer [11,12,13]. However, the potential role of adipose tissue–based imaging biomarkers in predicting pathologic complete response (pCR) remains poorly defined. In this context, the present study aims to investigate the association between visceral and subcutaneous adipose tissue parameters on pre-treatment imaging and pCR following neoadjuvant CCRT, and to explore their utility as predictive imaging biomarkers in patients with locally advanced rectal cancer.

## 2. Materials and Methods

### 2.1. Patient Selection and Grouping

This study enrolled patients with locally advanced rectal cancer who underwent preoperative CCRT at our institution between January 2020 and December 2024. The study was approved by the Institutional Review Board (IRB) of Soonchunhyang University Cheonan Hospital (Approval No. SCHCA 2025-10-017) and was conducted in accordance with the Declaration of Helsinki (2013 revision) and its subsequent amendments. This study was conducted as a retrospective analysis using previously collected clinical data, and IRB approval was obtained prior to data collection. The requirement for informed consent was waived by the IRB owing to the retrospective nature of the study.

The inclusion criteria were as follows: patients who (1) received preoperative CCRT and completed all treatment, (2) subsequently underwent surgery with definitive pathological findings, (3) had an Eastern Cooperative Oncology Group performance status of 0 or 1, (4) underwent non-contrast abdominal computed tomography (CT) before initiation of treatment, and (5) had no evidence of distant metastasis at diagnosis. The exclusion criteria were as follows: Patients who (1) did not complete preoperative CCRT or did not undergo surgery despite completing CCRT, (2) underwent surgery but had indeterminate pathological findings, (3) lacked pre-treatment non-contrast abdominal CT, or (4) had concomitant malignancies.

Based on these criteria, a total of 61 patients were included and analyzed. According to postoperative pathological treatment response, patients were classified into four subgroups: complete response (CR), near-complete response (near-CR), partial response (PR), and no response. For subgroup analysis, patients with CR and near-CR were grouped into the favorable response group, whereas those with PR and no response were grouped into the unfavorable response group. In addition, patients were reclassified into a response group (CR, near-CR, and PR) and a no-response group for further analysis.

### 2.2. Neoadjuvant Ccrt and Surgery

All 61 patients successfully completed neoadjuvant CCRT without any treatment interruptions. Concurrent CTx consisted of either oral capecitabine or intravenous 5-fluorouracil (5-FU) in all cases, and there were no missed administrations during the 25–28 fractions of radiotherapy (RT).

RT was performed as follows: all patients underwent simulation CT with a 3 mm slice thickness using a Philips Brilliance Big Bore scanner (Philips Medical Systems, Madison, WI, USA). Simulation was performed in the prone position to minimize small bowel irradiation. Treatment planning was conducted with either intensity-modulated radiotherapy (IMRT) or volumetric-modulated arc therapy (VMAT). The whole pelvis was irradiated with either 45 Gy in 25 fractions or 46 Gy in 23 fractions, followed by a tumor boost of 5.4 Gy in 3 fractions or 4 Gy in 2 fractions, resulting in a total dose of 50.4 Gy in 28 fraction or 50 Gy/25 fractions. All treatment plans were delivered using 6-MV photon beams and generated with Eclipse version 16.01.10 (Varian Medical Systems, Palo Alto, CA, USA).

Surgery was performed approximately two months after completion of neoadjuvant CCRT. All patients underwent definitive surgical procedures, including low anterior resection (LAR), ultra-low anterior resection (uLAR), or abdominoperineal resection (APR).

### 2.3. Postoperative Pathological Examination

Postoperative surgical specimens were evaluated by a pathologist with several decades of clinical experience. Pathologic tumor regression was assessed according to the Ryan tumor regression grading system, as adopted by the American Joint Committee on Cancer (AJCC) [14].

Tumor regression was graded as follows: grade 0, no viable cancer cells (complete response, CR); grade 1, single cells or rare small groups of cancer cells (near-complete response, near-CR); grade 2, residual cancer with evident tumor regression (partial response, PR); and grade 3, extensive residual cancer with no evident tumor regression (poor or no response). Based on this grading system, patients were classified into four subgroups: CR (grade 0), near-CR (grade 1), PR (grade 2), and no response (grade 3), and all subsequent analyses were performed according to these predefined groups.

### 2.4. Visceral, Subcutaneous Adipose Tissue and Body Composition Assessment

All body composition parameters were extracted from non-contrast-enhanced abdominal CT scans obtained in the supine position. Measurements were performed on a single axial slice at the level of the spinous process of the third lumbar vertebra (L3) [15].

Visceral adipose tissue (VAT) was defined using a CT attenuation (Hounsfield unit, HU) threshold range of −150 to −50 HU, while subcutaneous adipose tissue (SAT) was defined using a range of −190 to −30 HU [16]. Automated segmentation was performed using Eclipse ver. 16.01.10 (Varian Medical Systems, Palo Alto, CA, USA). In cases where non-adipose structures such as abdominal musculature or fecal material were included in the automated segmentation, manual correction was performed by a single experienced radiation oncologist who was blinded to all clinical and pathological data.

VAT-related parameters included VAT area (cm^2^), VAT index (VATI), calculated as VAT area (cm^2^) divided by height squared (m^2^), and mean VAT attenuation. Correspondingly, SAT area (cm^2^), SAT index (SATI), calculated as SAT area (cm^2^) divided by height squared (m^2^), and mean SAT attenuation were also measured [15].

In addition, pre-treatment body mass index (BMI = body weight (kg)/height^2^ (m^2^)) was calculated and included as a conventional anthropometric parameter for comparison with CT-based body composition indices [15].

### 2.5. Statistical Analyses

Continuous variables, including VAT-related parameters (VAT area, mean VAT attenuation, and VATI), SAT-related parameters (SAT area, mean SAT attenuation, and SATI), and BMI, were expressed as mean ± standard deviation and compared between groups using the independent samples *t*-test. Categorical variables were presented as frequencies and percentages and compared using the chi-square test or Fisher’s exact test, as appropriate. Receiver operating characteristic (ROC) curve analysis was performed to evaluate the predictive performance of adipose tissue parameters for pathologic treatment response. The area under the ROC curve (AUC) and its 95% confidence interval (CI) were calculated to quantify discriminative ability, with the CI estimated using the DeLong method. Optimal cutoff values were determined using the Youden index. Corresponding sensitivity, specificity, and accuracy were calculated for each optimal threshold.

All statistical analyses were performed using IBM SPSS Statistics (version 27.0; IBM Corp., Armonk, NY, USA). All tests were two-sided, and a *p*-value < 0.05 was considered statistically significant.

## 3. Results

### 3.1. Patient Characteristics

A total of 61 patients with rectal cancer who underwent neoadjuvant CCRT followed by surgery at our institution between 2020 and 2024 were included in the analysis. The mean age was 63.7 years (range, 26–86 years), 47 patients (77%) were male, and the mean body mass index (BMI) was 22.79 kg/m^2^. Most patients received a radiation fraction size of 200 cGy (77%), and the mean total radiation dose was 5009.18 cGy. The majority of patients had clinical stage IIIB disease (43.3%), followed by stage IIA (29.5%) and stage IIIC (20.0%). LAR was the most commonly performed surgery (83.6%), and the mean interval between completion of RT and surgery was 61.95 days. Baseline patient characteristics and CT-derived body composition parameters are summarized in Table 1 and Table 2, respectively.

### 3.2. Adipose Tissue Parameters Affecting Favorable Treatment Response

**Figure 1:**
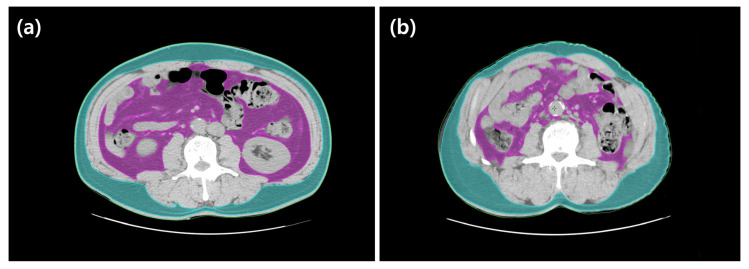
Representative computed tomography scans at the L3 level with identical body mass index (BMI) but varying visceral adipose tissue (VAT) area. (**a**) A patient with a BMI of 25.4 kg/m^2^ and a VAT area of 72.38 cm^2^, demonstrating a complete response. (**b**) A patient with an identical BMI of 25.4 kg/m^2^ and a VAT area of 16.82 cm^2^, demonstrating a partial response.

**Figure 2:**
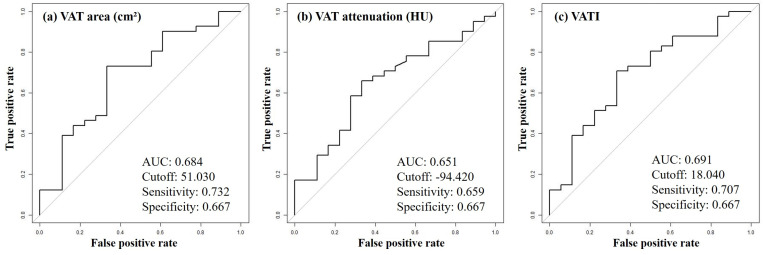
Receiver operating characteristic (ROC) curves of visceral adipose tissue (VAT)-related parameters for predicting favorable pathologic response. ROC curves are shown for (**a**) VAT area (cm^2^), (**b**) mean VAT attenuation (Hounsfield unit, HU), and (**c**) VAT index (VATI). All three VAT-related parameters were associated with favorable pathologic response.

To evaluate factors associated with pathologic response, patients with complete response (CR) or near-complete response (near-CR) were classified into the favorable response group (*n* = 18), while those with partial response (PR) or no response were classified into the unfavorable response group (*n* = 43). Factors significantly associated with pathologic response included BMI and VAT-related parameters, including mean VAT attenuation, VAT area, and VAT index (VATI). Compared with the unfavorable response group, the favorable response group demonstrated significantly higher BMI, lower mean VAT attenuation, larger VAT area, and higher VATI (Figure 1; Table 3). In contrast, SAT-related parameters, including mean SAT attenuation, SAT area, and SAT index (SATI) were not significantly associated with pathologic response. Additionally, initial clinical stage and the interval between completion of neoadjuvant CCRT and surgery were not associated with favorable pathologic response. ROC curve analysis was performed to evaluate the predictive performance of visceral adipose tissue-related parameters for favorable pathologic response. Among VAT-related variables, VATI demonstrated the highest discriminative ability, with an AUC of 0.691 (95% CI, [0.541–0.841]). The optimal cutoff value for VATI was 18.040, yielding a sensitivity of 70.7% and a specificity of 66.7%. The ROC curves of VAT-related parameters are presented in Figure 2.

### 3.3. Adipose Tissue Parameters Affecting the Absence of Treatment Response

**Figure 3:**
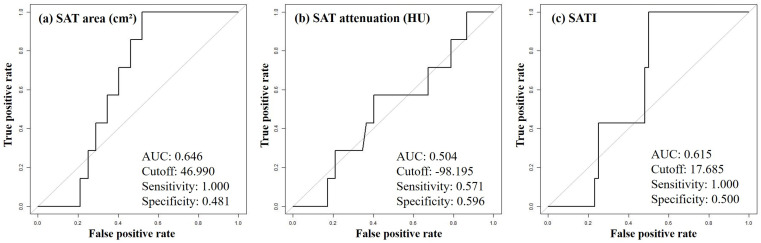
Receiver operating characteristic (ROC) curves of subcutaneous adipose tissue (SAT)-related parameters for predicting pathologic response. ROC curves were conducted for (**a**) SAT area (cm^2^), (**b**) mean SAT attenuation (Hounsfield unit, HU), and (**c**) SAT index (SATI). Among SAT-related parameters, all except mean SAT attenuation were significantly associated with pathologic response.

However, when patients were reclassified into a response group (CR, near-CR, and PR; *n* = 54) and a no-response group (*n* = 7), SAT-related parameters showed significant associations with treatment response. Specifically, SAT area and SATI, but not mean SAT attenuation, were significantly associated with response status (Table 4). In this analysis, VAT-related parameters, BMI, initial clinical stage, and the interval between completion of neoadjuvant CCRT and surgery were not significantly associated with pathologic response status. The ROC curves of SAT-related parameters are presented in Figure 3.

### 3.4. Prognostic Factors Affecting Cr and Near-Cr

Additionally, when analyzing the differences between the CR (*n* = 11) and near-CR (*n* = 7) groups, a difference in the CCRT schedule was observed. The mean total radiation dose was 5014.55 ± 20.18 (cGy) in the CR group, whereas it was 5000.00 ± 0.00 (cGy) in the near-CR group, showing a statistically significant difference (*p* = 0.038). However, in contrast to previous continuous analysis, no statistically significant differences were observed between the two RT dose schemes when analyzed as categorical variables. Also, no significant differences were observed in the type of concurrent CTx (oral capecitabine versus intravenous 5-fluorouracil) or in the administration schedule between the two groups (Table 5).

## 4. Discussion

Although adipose tissue comprises multiple anatomical depots, visceral and subcutaneous adipose tissues represent two biologically distinct compartments with different clinical implications. VAT, located within the abdominal cavity, is metabolically active and characterized by a pro-inflammatory phenotype and increased lipolytic activity. In contrast, SAT, situated beneath the skin, is comparatively less metabolically active and primarily serves as an energy reservoir with a relatively protective metabolic role. These fundamental differences suggest that VAT and SAT may differentially influence tumor biology and treatment response [17,18].

VAT has been widely implicated in carcinogenesis through chronic inflammation, insulin resistance, and adipokine dysregulation [19,20]. In the context of active cancer treatment, radiation-induced immunogenic cell death may alter the tumor microenvironment toward a more immunogenic state. In this setting, VAT-derived cytokines and adipokines may be associated with biological conditions that could influence treatment response [21,22,23]. In addition, VAT-associated angiogenic factors, such as vascular endothelial growth factor (VEGF) and interleukin-8 (IL-8), may contribute to improved tumor oxygenation and potentially enhance radiosensitivity [24,25,26]. These findings may provide a potential biological explanation for the observed association between higher VAT and favorable treatment response and may be consistent with previous reports suggesting a similar association [27]. However, these interpretations remain speculative, as the present study did not include tissue-level analyses or systemic cytokine profiling. Further translational studies are warranted to elucidate the underlying biological mechanisms.

Importantly, the present study further suggests that not only the quantity but also the qualitative characteristics of VAT are relevant to treatment response. Mean VAT attenuation on CT reflects tissue composition, with low attenuation values indicating higher lipid content and functionally preserved adipose tissue, whereas higher HU values reflect inflammation, fibrosis, or increased water content [28]. The finding that favorable responders exhibited both larger VAT area and lower mean VAT attenuation suggests that metabolically intact, lipid-rich VAT may be associated with biological conditions that could potentially support pro-immunogenic and pro-apoptotic processes during treatment. To our knowledge, this is among the first studies to highlight the potential role of adipose tissue quality, as assessed by CT attenuation, in predicting pathologic response to neoadjuvant CCRT in rectal cancer.

In contrast to these findings, SAT is situated between the skin and the fascial layer and is comparatively less metabolically active, serving a relatively protective role. SAT functions primarily as an energy reservoir and plays a key role in thermoregulation [17,18]. Reduced SAT may represent an early manifestation of cachexia, a state in which systemic metabolic depletion renders patients more vulnerable to nutritional deterioration and declining performance status during the course of treatment [13,15]. Moreover, cachexia-associated SAT loss has been implicated in the impairment of the tumor microenvironment’s immune function, potentially compromising the body’s capacity to clear treatment-damaged tumor cells [15]. Notably, unlike VAT, whose metabolic activity and cytokine secretory capacity render its qualitative characteristics functionally relevant, SAT operates predominantly as a passive energy depot, suggesting that its quantity rather than its quality may be the primary determinant of its functional contribution. This distinction may partly explain why SAT area and SATI, but not mean SAT HU, were significantly associated with treatment response in the present study, and may further supports the notion that visceral and subcutaneous adipose tissue depots exert their influence on treatment outcomes through fundamentally different mechanisms. However, these findings should be interpreted with caution, as the number of patients in the no-response group was limited, which may increase the risk of statistical instability and potential artifacts. Therefore, the observed association between SAT parameters and treatment response should be considered exploratory and hypothesis-generating, requiring validation in larger cohorts.

In the present study, CT attenuation demonstrated different clinical implications depending on the type of adipose tissue compartment; however, the qualitative characteristics of adipose tissue, as reflected by CT attenuation, may nonetheless carry prognostic significance in other clinical contexts. Emerging evidence suggests that CT-derived adipose tissue attenuation may provide clinically relevant information beyond fat quantity alone. In patients with hepatocellular carcinoma, higher SAT density has been independently associated with worse survival, potentially reflecting adipose tissue inflammation, fibrosis, or altered lipid composition [28]. In the present study, mean VAT attenuation was significantly associated with favorable treatment response, whereas mean SAT attenuation was not associated with treatment response in the response versus no-response analysis. These findings suggest that the mechanism by which adipose tissue influences treatment outcomes may differ depending on the type of malignancy.

In the present study, BMI was associated with favorable pathologic response. However, this association was not consistently observed across different response classifications. This variability suggests that while BMI may provide a general estimate of overall adiposity, its predictive value may be limited in this context. Importantly, BMI does not distinguish between visceral and subcutaneous adipose tissue depots and does not capture qualitative differences in adipose tissue composition. Prior studies have shown that CT-derived adipose tissue parameters more accurately reflect metabolic vulnerability and clinical outcomes in cancer patients, suggesting that CT-based body composition analysis may provide a more refined assessment of metabolic and inflammatory status than conventional anthropometric measures such as BMI [15,17].

Beyond adipose tissue parameters, a subgroup analysis comparing the CR and near-CR groups revealed several noteworthy findings. No significant differences were observed in CTx-related factors, including regimen and administration schedule. In contrast, RT-related parameters, including total radiation dose, fraction size, and number of fractions, showed statistically significant associations with response depth when analyzed as continuous variables; however, these associations were not maintained in categorical analyses. This discrepancy may be attributable to the limited sample size, reduced statistical power following categorization, and the relatively narrow range of RT protocols in this cohort. Although modest differences in total radiation dose, fraction size, and number of fractions were observed between groups, it remains uncertain whether these variations have clinically meaningful effects on the depth of tumor regression. Accordingly, these findings should be interpreted with caution, and further studies with larger sample sizes and more heterogeneous RT regimens are warranted to clarify these associations.

This study has several limitations. The cohort was relatively small and derived from a single institution, and the number of patients in certain subgroups, particularly the no-response group, was limited, which may have reduced the statistical robustness of subgroup analyses and increased the risk of type I error.

The mean BMI of the study population was within the normal to mildly overweight range, with no patients meeting the criteria for severe obesity or underweight according to Korean standards. Therefore, the generalizability of these findings to populations across the BMI spectrum may be limited, and further validation in larger and more diverse cohorts is warranted.

## 5. Conclusions

In conclusion, visceral and subcutaneous adipose tissue parameters derived from CT imaging were differentially associated with pathologic response to neoadjuvant CCRT in locally advanced rectal cancer. These findings suggest that adipose tissue is not merely a passive energy depot, but a biologically active component that may influence treatment response in a compartment-specific manner. These findings indicate that CT-based body composition analysis may provide clinically relevant information beyond conventional measures such as BMI and may serve as a potential imaging biomarker for predicting treatment response. Further large-scale prospective studies are warranted to validate these findings and to explore their integration into clinical decision-making.

## Figures and Tables

**Table 1:** Patients characteristics (*n* = 61).

	*n* (% or Range)
Mean age	63.70 (26–86)
Sex	
Male	47 (77.0)
Female	14 (23.0)
Fraction size (cGy)	
180	14 (23.0)
200	47 (77.0)
Total radiation dose (cGy)	5009.18 (5000.0–5040.0)
Clinical Stage	
IIA *	18 (29.5)
IIB	3 (5.0)
IIIA	1 (1.7)
IIIB	26 (43.3)
IIIC	12 (20.0)
Surgery name	
LAR	51 (83.6)
APR	5 (8.2)
Ultra LAR	5 (8.2)
Interval from RT completion to surgery (days)	61.95 (42.0–132.0)
Height (cm)	162.86 (139.0–181.0)
Weight (kg)	60.68 (34.2–84.0)
BMI (kg/m^2^)	22.79 (15.2–29.9)

Abbreviations: LAR, low anterior resection; APR, abdominoperineal resection; RT, radiotherapy; BMI, body mass index; * Patients with stage IIA disease included those with high-risk features.

**Table 2:** CT-derived body composition parameters (*n* = 61).

	Mean ± SD
VAT area (cm^2^)	46.97 ± 43.04
SAT area (cm^2^)	50.85 ± 36.41
mean VAT attenuation (HU)	−90.85 ± 9.04
mean SAT attenuation (HU)	−93.40 ± 10.28
VATI (cm^2^/m^2^)	17.67 ± 15.91
SATI (cm^2^/m^2^)	19.87 ± 15.78

Abbreviations: VAT, visceral adipose tissue; SAT, subcutaneous adipose tissue; HU, Hounsfield unit; VATI, visceral adipose tissue index; SATI, subcutaneous adipose tissue index.

**Table 3:** CT-derived adipose tissue parameters affecting favorable and unfavorable groups.

	Favorable Group (n = 18)	Unfavorable Group (n = 43)	*p*-Value
BMI (kg/m^2^)	24.39 ± 2.98	22.12 ± 3.31	0.015
VAT_area (cm^2^)	67.71 ± 54.15	37.86 ± 34.08	0.013
mean VAT attenuation (HU)			0.033
high HU (−50~−94)	6 (33.3%)	26 (63.4%)	
Low HU (−95~−150)	12 (66.7%)	15 (36.6%)	
VATI (cm^2^/m^2^)	25.14 ± 18.95	14.39 ± 13.35	0.015
SAT_area (cm^2^)	59.66 ± 33.70	46.98 ± 37.28	0.221
mean SAT attenuation (HU)			0.181
high HU (−30~−99)	9 (50%)	13 (31.7%)	
Low HU (−100~−190)	9 (50%)	28 (68.3%)	
SATI (cm^2^/m^2^)	23.25 ± 14.95	18.39 ± 16.09	0.280

Abbreviations: CT, computed tomography; BMI, body mass index; VAT, visceral adipose tissue; HU, Hounsfield unit; VATI, visceral adipose tissue index; SAT, subcutaneous adipose tissue; SATI, subcutaneous adipose tissue index.

**Table 4:** CT-derived adipose tissue parameters affecting response and no-response groups.

	Response Group (*n* = 54)	No-Response Group (*n* = 7)	*p*-Value
BMI (kg/m^2^)	22.84 ± 3.51	22.41 ± 1.94	0.751
VAT_area (cm^2^)	47.47 ± 44.11	43.21 ± 36.71	0.808
mean VAT attenuation (HU)			0.692
high HU (−50~−94)	29 (55.8%)	3 (42.9%)	
Low HU (−95~−150)	23 (44.2%)	4 (57.1%)	
VATI (cm^2^/m^2^)	17.87 ± 16.35	16.19 ± 13.06	0.796
SAT_area (cm^2^)	53.10 ± 38.12	34.11 ± 9.81	0.006
mean SAT attenuation (HU)			>0.999
high HU (−30~−99)	19 (36.5%)	3 (42.9%)	
Low HU (−100~−190)	33 (63.5%)	4 (57.1%)	
SATI (cm^2^/m^2^)	20.74 ± 16.58	13.39 ± 4.13	0.012

Abbreviations: CT, computed tomography; BMI, body mass index; VAT, visceral adipose tissue; HU, Hounsfield unit; VATI, visceral adipose tissue index; SAT, subcutaneous adipose tissue; SATI, subcutaneous adipose tissue index.

**Table 5:** Factors associated with CR versus near-CR.

	CR Group (*n* = 11)	Near-CR Group (*n* = 7)	*p*-Value
BMI (kg/m^2^)	24.17 ± 2.37	24.73 ± 3.95	0.709
VAT_area (cm^2^)	60.41 ± 50.90	79.18 ± 61.13	0.490
mean VAT attenuation (HU)			>0.999
high HU (−50~−94)	4 (36.4%)	2 (28.6%)	
Low HU (−95~−150)	7 (63.6%)	5 (71.4%)	
VATI (cm^2^/m^2^)	22.51 ± 16.84	29.27 ± 22.63	0.477
SAT_area (cm^2^)	52.49 ± 24.47	70.92 ± 44.47	0.271
mean SAT attenuation (HU)			>0.999
high HU (−30~−99)	5 (45.5%)	4 (57.1%)	
Low HU (−100~−190)	6 (54.5%)	3 (42.9%)	
SATI (cm^2^/m^2^)	20.96 ± 11.97	26.85 ± 19.23	0.432
RT scheme			
Fraction size (cGy)	192.73 ± 10.09	200.00 ± 0.00	0.038
Fractions	26.09 ± 1.51	25.00 ± 0.00	0.038
Total RT dose (cGy)	5014.55 ± 20.18	5000.00 ± 00	0.038
Fraction size			0.119
180 cGy	4 (36.4%)	0 (0.0%)	
200 cGy	7 (63.6%)	7 (100.0%)	
Fractions			0.119
28 fractions	4 (36.4%)	0 (0.0%)	
25 fractions	7 (63.6%)	7 (100.0%)	
Total RT dose			0.119
5040 cGy	4 (36.4%)	0 (0.0%)	
5000 cGy	7 (63.6%)	7 (100.0%)	
CTx scheme			>0.999
Oral capecitabine	7 (63.6%)	5 (71.4%)	
Intravenous 5-FU	4 (36.4%)	2 (28.6%)	

Abbreviations: CR, complete response; BMI, body mass index; VAT, visceral adipose tissue; HU, Hounsfield unit; VATI, visceral adipose tissue index; SAT, subcutaneous adipose tissue; SATI, subcutaneous adipose tissue index; CTx, chemotherapy; 5-FU, 5-fluorouracil.

## Data Availability

The data presented in this study are available from the corresponding author upon reasonable request. The data are not publicly available due to privacy and ethical restrictions.

## Abbreviations

BMI	body mass index
CCRT	concurrent chemoradiotherapy
CR	complete response
CT	computed tomography
CTx	chemotherapy
IMRT	intensity-modulated radiotherapy
near-CR	near-complete response
pCR	pathologic complete response
PR	partial response
RT	radiotherapy
SAT	subcutaneous adipose tissue
SATI	subcutaneous adipose tissue index
TNT	total neoadjuvant therapy
VAT	visceral adipose tissue
VATI	visceral adipose tissue index
VMAT	volumetric-modulated arc therapy
